# Case Report: Hidden B-cell dysregulation in MIRAGE syndrome

**DOI:** 10.3389/fped.2026.1765896

**Published:** 2026-04-10

**Authors:** Shulan Zhang, Feng Liu, Xia Huang

**Affiliations:** Department of Respiratory Medicine, Children’s Hospital of Nanjing Medical University, Nanjing, China.

**Keywords:** B cell, immune cells, immunodeficiency, MIRAGE syndrome, SAMD9

## Abstract

**Aim:**

To characterize the immune and molecular abnormalities underlying MIRAGE syndrome by profiling peripheral blood transcriptomes and assessing immune cell composition and function in affected patients.

**Methods:**

We performed RNA sequencing on peripheral whole-blood samples collected from identical twins diagnosed with MIRAGE syndrome and their healthy parents. Bulk RNA-seq data were subsequently deconvoluted to assess the composition, state, and functional characteristics of immune cell populations.

**Results:**

Differentially expressed genes between patients and healthy parents were significantly enriched in the PI3K–Akt signaling pathway, B-cell receptor signaling pathway, and primary immunodeficiency-related pathways. Patients exhibited reduced proportions of memory and naïve B cells, accompanied by increased proportions of CD8 T cells and M2 macrophages. We further identified the top 10 hub genes, which showed moderate to strong correlations with B-cell differentiation, proliferation, and activation.

**Conclusion:**

Immune cell dysregulation is evident in patients with MIRAGE syndrome, with B-cell abnormalities representing a prominent immunological feature.

## 1 Introduction

MIRAGE syndrome is a recently described multisystem genetic disorder characterized by myelodysplasia, infections, growth restriction, adrenal hypoplasia, genital phenotypes, and enteropathy, and is caused by gain-of-function mutations in the SAMD9 gene (1). This gene is located on the long arm of chromosome 7 at position 7q21.2 and encodes sterile alpha motif domain-containing protein 9 (SAMD9), which acts as a growth repressor (2). The mutation leads to a multisystem disorder characterized by organ hypoplasia due to impaired cellular proliferation (1–3). MIRAGE syndrome is associated with a high mortality rate, with most patients dying in infancy or early childhood (2). The majority of the patients present with recurrent pulmonary infections, which may be related to gastroesophageal reflux (4) and immunodeficiency (5, 6). However, relatively few studies have focused on the immune status and function of these patients.

Deconvolution of bulk RNA sequencing data can provide deeper insights into the alterations in immunological features and functions. In this study, performed RNA sequencing on peripheral whole blood samples collected from identical twin patients with MIRAGE syndrome and their healthy parents. We then deconvoluted the bulk sequencing data to evaluate the status of immune cells which may be affected by growth repressor. This analysis provides a foundation for investigating the mechanisms underlying MIRAGE syndrome and for implementing immune support therapies.

## 2 Method

### 2.1 Isolate peripheral leukocytes

Four milliliters of EDTA-anticoagulated whole blood were collected from identical twins with MIRAGE syndrome and their healthy parents. Leukocytes were isolated from the blood specimens using the RBC lysis method (Servicebio, G2015-500ML) according to the manufacturer's procedure. The isolated leukocytes were then immediately stored in 1 mL of Trizol reagent (Thermo Fisher, 15596018) and kept in a −80 °C freezer (7).

### 2.2 RNA extraction and sequencing analyses

Total RNA was extracted using Trizol reagent (Thermofisher, 15596018) following the manufacturer's procedure. The RNA libraries were sequenced on the illumina NovaseqTM 6000 platform by Biomarker Bio Technology CO., Ltd (Beijing, China). Total RNA was extracted and assessed for purity, concentration, and integrity using a NanoDrop 2000 spectrophotometer and an Agilent 2100 Bioanalyzer. Only samples meeting standard RNA quality criteria were used for library preparation. Poly(A)-tailed mRNA was enriched, fragmented, and reverse-transcribed into cDNA, followed by end repair, adapter ligation, size selection, and PCR amplification. Library quality and concentration were evaluated using a Qubit 3.0 fluorometer, Qsep400 system, and quantitative PCR. Libraries were sequenced on an Illumina NovaSeq 6000 platform (paired-end 150 bp) by Biomarker Bio Technology Co., Ltd. (Beijing, China). RNA sequencing was performed on four samples, generating a total of 25.15 Gb of clean data (approximately 6.16 Gb per sample). The percentage of bases with quality scores ≥ Q30 exceeded 95.35% across all samples. Clean reads were aligned to the reference genome with mapping rates ranging from 97.06% to 97.19%. Downstream transcriptomic analyses were conducted using the BMKCloud platform following standard pipelines. Due to limited sample availability, technical replicates were not included.

### 2.3 Differentially expressed genes (DEGs) and enrichment analyses

Differentially expressed genes (DEGs) analysis was performed by edgeR between two samples (8). |log_2_ fold change| > 1 and false discovery rate (FDR) < 0.05 were used to screen differential genes (DEGs). DEGs were then subjected to enrichment analyses of Gene Ontology (GO) functions and Kyoto Encyclopedia of Genes and Genomes (KEGG) pathways. Gene set enrichment analysis (GSEA) analysis of all genes was predominantly performed on KEGG pathways.

### 2.4 Protein–protein interaction (PPI) network construction and examination

To identify the hub genes, the STRING database (http://string-db.org) (version 12) was used to construct the PPI network, which was then visualized using Cytoscape software (version 3.9.1). The top 10 hub genes were identified using the cytoHubba plug-in in Cytoscape.

### 2.5 Evaluation of immune cells

We used the “CIBERSORTx” website (https://cibersortx.stanford.edu/) to estimate the fraction of 22 types of immune cells between patients and their healthy parents.

## 3 Results

### 3.1 Clinical features and genetic diagnosis

**Figure 1:**
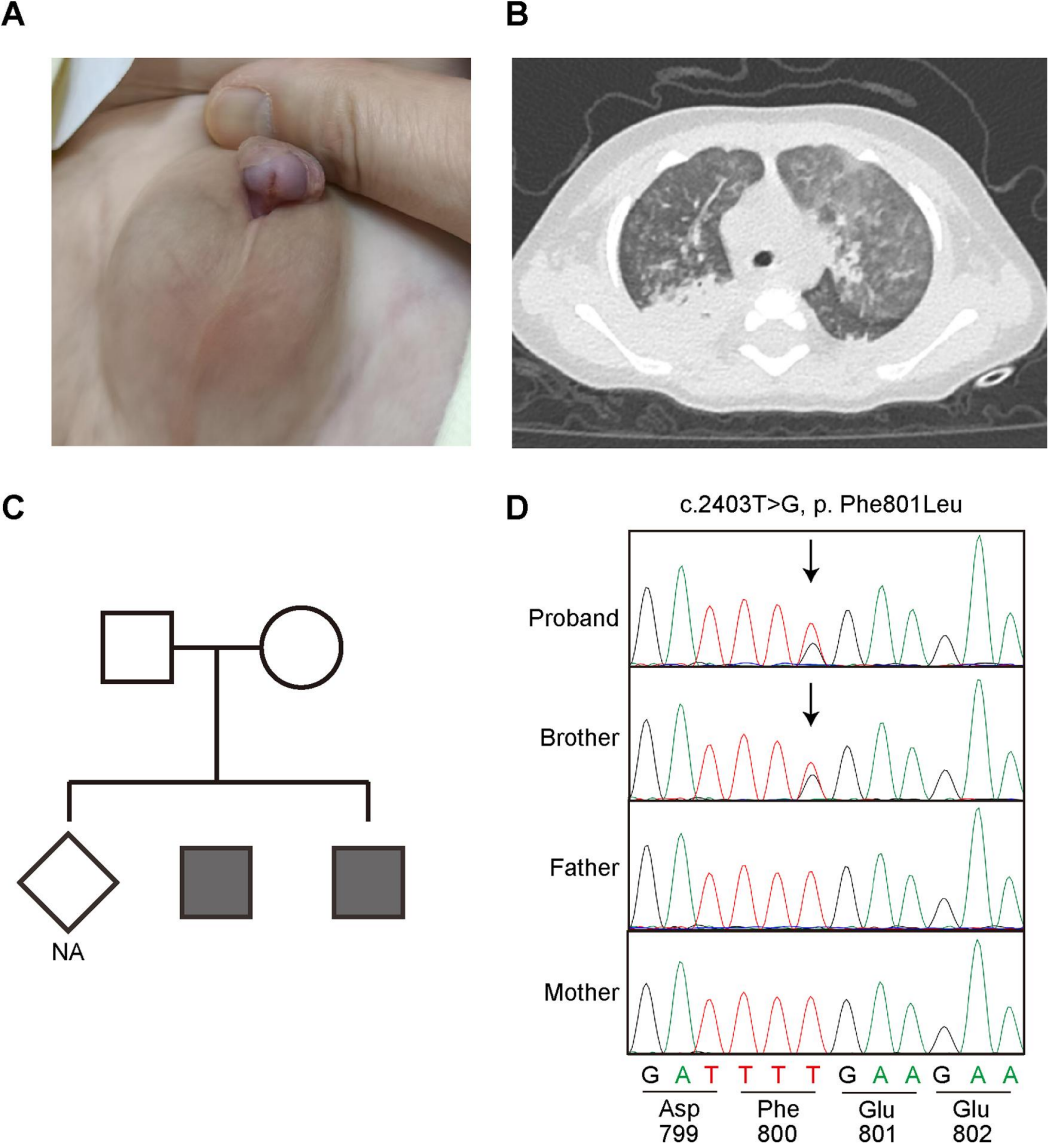
Clinical findings, family pedigree and variant screening analysis. The identical twin patients suffer from hypospadias **(A)** and recurrent lung infections **(B)** Pedigree of the patients is shown with SAMD9 genotype information **(C)** Dark symbols represent the patient affected by MIRAGE syndrome, and clear symbols represent the unaffected members. Rhombus indicate stillbirth. NA denotes genotype not available. A square indicates a male and a circle indicates a female. **(D)** The twin patients have a heterozygous missense variant (c.2403T > G, p. Phe801Leu) mutation in SAMD9. The arrowhead shows the mutated nucleotide.

This case involved male identical twins (proband and his brother) born at 34 weeks' gestation with a birth weight of 1,100 g and 1,200 g (Table 1 and Figure 1). They experienced hypoxia and required ventilator support. They also had pneumonia, anemia, necrotizing enterocolitis, bronchopulmonary dysplasia, and hypospadias. After birth, they suffered from chronic diarrhea and recurrent pulmonary infections and were unable to be weaned off oxygen. Multiple sputum cultures and bronchoalveolar lavage fluid cultures indicated infection with *Klebsiella pneumoniae*. We conducted an immunologic evaluation, which included lymphocyte subset enumeration and immunoglobulin tests (Table 1).

**Table 1:** Clinical characteristics of the patients.

Variables	Proband	Brother	Reference ranges
Chromosome karyotype	46XY	46XY	
SAMD9	c.2403T > G (p. Phe801Leu)	c.2403T > G (p. Phe801Leu)	
Gestation at Delivery （week）	34	34	
Intrauterine growth retardation	Yes	Yes	
Birth weight (Kg)	1.1	1.2	
Myelosuppression	NO	NO	
Infections	Recurrent pneumonia	Recurrent pneumonia	
Growth restriction	Yes	Yes	
Adrenal insufficiency	No	Yes	
Genitourinary malformation	hypospadias	hypospadias	
Enteropathy	Chronic diarrhea	Chronic diarrhea	
Immunoglobulin G (g/L)	18.8	5.34	3.05–6.87
Immunoglobulin M (g/L)	1. 1	0.65	0.31–0.85
Immunoglobulin A (g/L)	0.093	0.078	0.11–0.45
CD3^+^ (T cells) %	92.2	86.9	59–84
CD3 ^+^ CD4^+^ (CD4 ^+^ T) %	54.18	51.33	31–60
CD3 ^+^ CD 8 ^+^ (CD8 ^+^ T cell) %	37.93	34.45	13–38
CD3^−^CD16 ^+^ CD56^+^（NK cell) %	1.74	4.36	6–27
CD3^−^CD19^+^ (B cell)	4.98	7.93	7–22
Cortisol (nmol/L)	10.1	132.0	133–537
Adrenocorticotrophic hormone (pg/mL)	26.7	110.0	0–46

They showed recurrent infections (I), growth restriction(R), genital phenotypes (hypospadias) (G), and enteropathy (chronic diarrhea) (E), and adrenal insufficiency (A) (the older brother), although he did not suffer from myelosuppression (M). Whole-exome sequencing was performed at 7 months of age and identified a novel heterozygous missense variant in SAMD9 (c.2403T > G, p.Phe801Leu) in both twin patients (Figure 1). This variant has not been reported in public population databases. Given the established association between SAMD9 and MIRAGE syndrome, the clinical phenotype of the patients was considered consistent with an atypical presentation of MIRAGE syndrome.

### 3.2 DEGs and functional enrichment analysis

**Figure 2:**
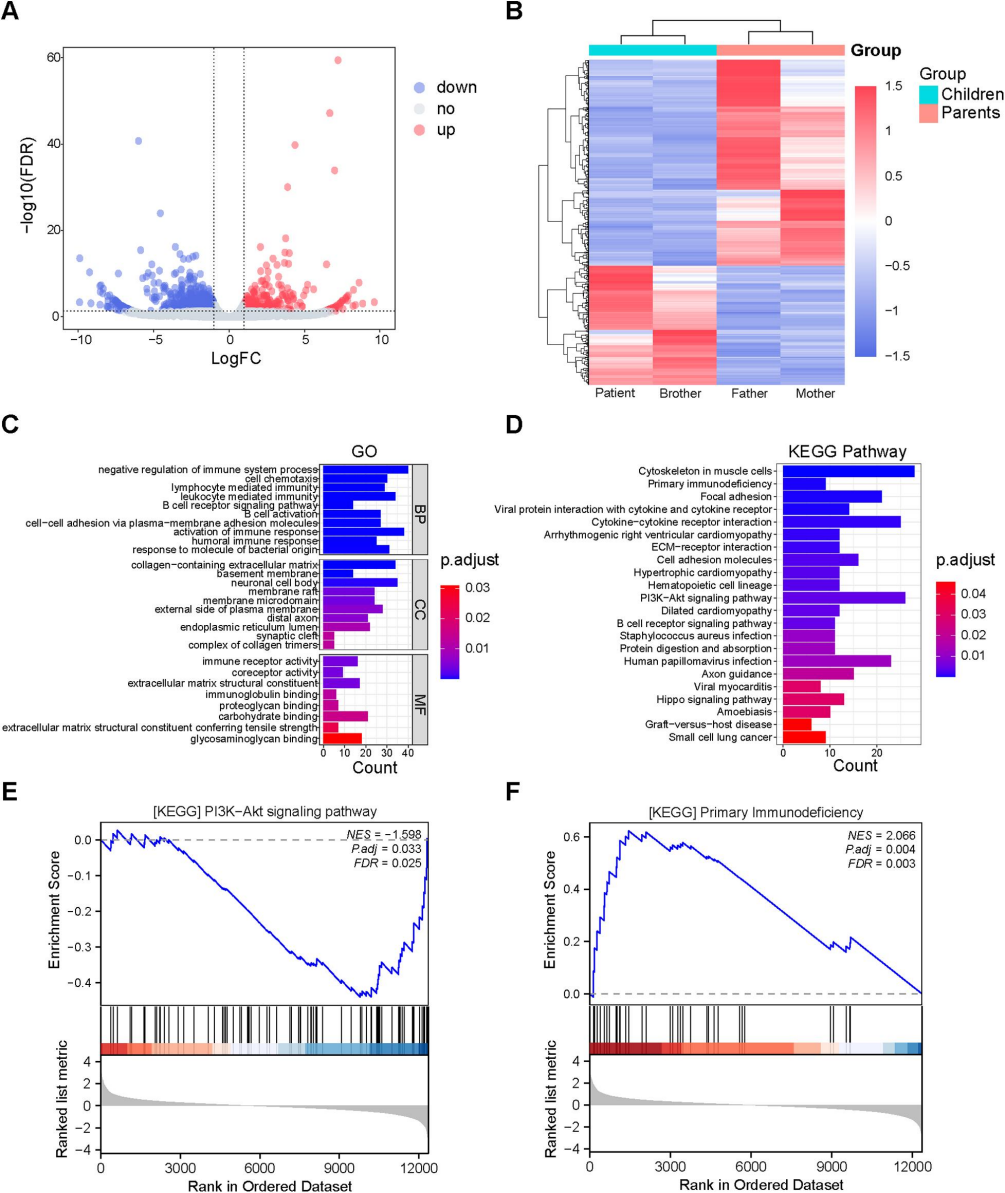
DEGs and functional enrichment analysis between children with MIRAGE syndrome and their patients. **(A)** Volcano map shows the Differentially expressed genes (DEGs)in children and parents, with red and blue dots indicating gene up-regulation and down-regulation, respectively. **(B)** Heat map of DEGs. Red represents up-regulated genes and blue represents down-regulated genes. GO **(C)** and KEGG **(D)** enrichment analysis of DEGs. Gene set enrichment analysis (GSEA) of all genes **(E,F)**.

A total of 663 DEGs were identified between the children and their parents, with 242 genes upregulated and 421 genes downregulated (Figures 2A,B). The DEGs were then analyzed using both Gene Ontology (GO) functional analysis and Kyoto Encyclopedia of Genes and Genomes (KEGG) pathway enrichment analysis to predict their potential biological functions. The most significant enrichment terms for GO were negative regulation of immune system process, activation of immune response, B cell activation (biological process), collagen−containing extracellular matrix, basement membrane and neuronal cell body (cellular component), immune receptor activity, coreceptor activity, and extracellular matrix structural constituent (molecular function) (Figure 2C). The DEG signaling pathways were primarily enriched in the PI3K-Akt pathway, B cell receptor signaling pathway, and primary immunodeficiency pathways (Figure 2D). The results of GSEA enrichment analysis indicated that the inhibition of the PI3K- Akt signaling pathway significantly enhanced the primary immunodeficiency pathways (Figures 2E,F).

### 3.3 Correlation between hub genes and immune cells

**Figure 3:**
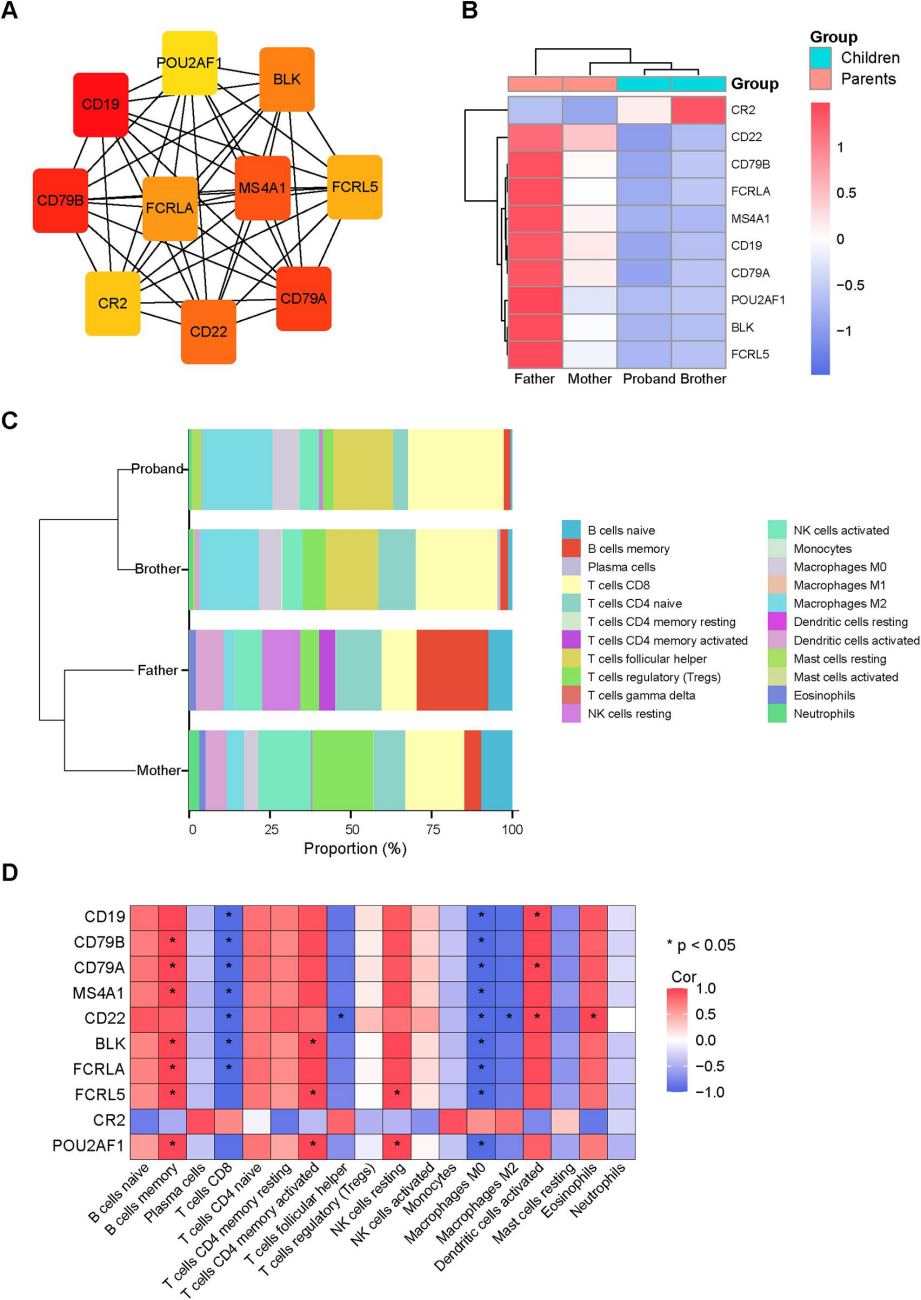
Correlation analyses of hub genes and immune cells. **(A)** The top 10 hub genes in the protein–protein interaction network. The darker the orange color, the higher the score. **(B)** Heatmaps showing the top 10 hub genes. Red represents up-regulated genes and blue represents down-regulated genes. **(C)** Estimated proportion of the immune cell subpopulations. **(D)** correlation heat map was used to show the correlation between the hub genes and immune cells. Red depicts a positive correlation, whereas blue indicates a negative correlation.

To identify the hub genes, the PPI network for the DEGs constructed and visualized using the STRING database and Cytoscape software, respectively. The top 10 hub genes were identified as follows: CD19, CD79B, CD79A, MS4A1, CD22, BLK, FCRLA, FCRL5, CR2, and POU2AF1. Compared to their parents, most of the hub genes in the patients were downregulated, with the exception of CR2. (Figures 3A,B).

To investigate the composition of immune cells in each sample, we used CIBERSORTx to deconvolution of bulk RNA-seq data. As shown in Figure 3C, the proportions of memory B cells, naïve B cells, and activated NK cells were reduced, while the proportions of CD8 T cells and M2 macrophages were increased in the patients. We then analyzed the associations between the hub genes and immune cells. As shown in Figure 3D, the top 10 hub genes exhibited moderate to strong correlations with memory B cells, CD8 T cells and M2 macrophages.

## 4 Discussion

Here, we attempted to use bulk RNA sequencing to assess the status and function of immune cells in identical twin patients with MIRAGE syndrome. The GO function enrichment analysis showed that DEGs were primarily involved in the negative regulation of immune system processes, activation of immune responses, and B cell activation. The DEG signaling pathways were significantly enriched in the PI3K-Akt pathway, B cell receptor signaling pathway, and primary immunodeficiency pathways. The phosphoinositide 3-kinase (PI3 K) cascade is a widely expressed signal transduction pathway that plays a crucial role in promoting cell survival, proliferation, and metabolism (9). Studies have shown that knocking down SAMD9 in glioma cells decreases glioblastoma progression via the PI3K-Akt pathway (10, 11); however, whether this pathway is involved in immune regulation in the context of MIRAGE syndrome remains unknown. Therefore, we speculate that the mutated SAMD9 may inhibit the proliferation and activity of immune cells through the PI3K-Akt signaling pathway.

There have been few studies on the evaluation of immune cell functions in this disease. The limited studies available show that patients have reduced B cells, slightly lower levels of IgG, normal levels of IgA and IgM, but a lack of specific antibodies. This was diagnosed based on poor specific antibody response to pneumococcal polysaccharide antigens (5, 6). Notably, immunoglobulin replacement therapy has been reported to improve clinical outcomes in affected individuals, supporting the presence of functional humoral immune deficiency (5). SAMD9 could enhance the infiltration ability of M2 macrophage cells (11). Consistent with these earlier studies, our research found that the proportion of B cells, including both memory and naïve B cells, was decreased, while the levels of CD8 T cells and M2 macrophages were elevated. The top 10 hub genes are also related to B cell development and activation, and the expression of the vast majority of them is decreased. These findings indicate a more pronounced suppression of B cell proliferation, which in turn leads to a compensatory increase in T cells among patients with MIRAGE syndrome.

This study has several important limitations. First, the sample size is extremely small, consisting of two affected individuals from a single family, which inherently limits statistical power. Second, patients with MIRAGE syndrome often experience severe infections, hypoxia, prematurity, prolonged oxygen therapy, and extensive antibiotic exposure, all of which are known to independently affect immune gene expression profiles. Given the limited sample size and the complex clinical background of the affected individuals, it was not possible to control for or disentangle the effects of these potential confounders. Therefore, the immune-related findings reported here should be interpreted as descriptive transcriptional associations rather than disease-specific causal mechanisms. In addition, functional studies will be required to clarify the pathogenic mechanism of the identified *SAMD9* variant and its contribution to phenotypic variability in MIRAGE syndrome.

In conclusion, we have documented the clinical characteristics of identical twin patients with MIRAGE syndrome and performed RNA sequencing analysis on the peripheral blood of both the patients and their parents. Our findings suggest that B cell function in the children is inhibited. However, further research is needed to corroborate this observation. Nonetheless, our study lays the groundwork for potential immunological supportive treatments.

## Data Availability

The datasets presented in this study can be found in online repositories. The names of the repository/repositories and accession number(s) can be found in the article/Supplementary Material.
